# Genome-Wide Association Study of Calcium Accumulation in Grains of European Wheat Cultivars

**DOI:** 10.3389/fpls.2017.01797

**Published:** 2017-10-27

**Authors:** Dalia Z. Alomari, Kai Eggert, Nicolaus von Wirén, Klaus Pillen, Marion S. Röder

**Affiliations:** ^1^Leibniz Institute of Plant Genetics and Crop Plant Research, Gatersleben, Germany; ^2^Institute of Agricultural and Nutritional Sciences, Martin Luther University Halle-Wittenberg, Halle, Germany

**Keywords:** wheat, calcium, GWAS, MTAs, mineral concentration

## Abstract

Mineral concentrations in cereals are important for human health, especially for people who depend mainly on consuming cereal diet. In this study, we carried out a genome-wide association study (GWAS) of calcium concentrations in wheat (*Triticum aestivum* L.) grains using a European wheat diversity panel of 353 varieties [339 winter wheat (WW) plus 14 of spring wheat (SW)] and phenotypic data based on two field seasons. High genotyping densities of single-nucleotide polymorphism (SNP) markers were obtained from the application of the 90k iSELECT ILLUMINA chip and a 35k Affymetrix chip. Inductively coupled plasma optical emission spectrometry (ICP-OES) was used to measure the calcium concentrations of the wheat grains. Best linear unbiased estimates (BLUEs) for calcium were calculated across the seasons and ranged from 288.20 to 647.50 among the varieties (μg g^-1^ DW) with a mean equaling 438.102 (μg g^-1^ DW), and the heritability was 0.73. A total of 485 SNP marker–trait associations (MTAs) were detected in data obtained from grains cultivated in both of the two seasons and BLUE values by considering associations with a -log_10_ (*P*-value) ≥3.0. Among these SNP markers, we detected 276 markers with a positive allele effect and 209 markers with a negative allele effect. These MTAs were found on all chromosomes except chromosomes 3D, 4B, and 4D. The most significant association was located on chromosome 5A (114.5 cM) and was linked to a gene encoding cation/sugar symporter activity as a potential candidate gene. Additionally, a number of candidate genes for the uptake or transport of calcium were located near significantly associated SNPs. This analysis highlights a number of genomic regions and candidate genes for further analysis as well as the challenges faced when mapping environmentally variable traits in genetically highly diverse variety panels. The research demonstrates the feasibility of the GWAS approach for illuminating the genetic architecture of calcium-concentration in wheat grains and for identifying putative candidate genes underlying this trait.

## Introduction

Hexaploid wheat (*Triticum aestivum* L.) is one of the most essential and widely planted crops worldwide with its products feeding most of the global population (FAO, 2016)^1^. Many people relying strongly on wheat-based food stuff suffer from nutrient deficiencies, especially of Fe, Zn, Ca, and Mg (Welch and Graham, 2004; White and Broadley, 2005, 2009; Yano et al., 2016), because wheat grains contain low amounts of these nutrients. Genetic biofortification is one strategy involving plant breeding, which offers a sustainable and long-term approach for developing mineral-rich crop varieties (Bouis, 2007; Velu et al., 2014). This requires a better understanding of the genetic basis of mineral element accumulation in wheat grains that improves wheat quality and its value for human dietary consumption. Calcium plays an important role in cell wall structure, plant architecture, quality, and yield formation, while its deficiency makes the plant more sensitive to biotic and abiotic stresses (Dayod et al., 2010). Most of calcium dietary consumption in humans is lower than the recommended daily intake (RDI) of 800–1,300 mg per capita (Kranz et al., 2007). Adequate calcium intake especially during adolescence is critical to reduce the rate of bone loss, rickets, and osteoporosis, while lower intake provokes health risks, such as hypocalcemia, hypertension, colorectal cancer as well as bone weakness and fractures accompanied with aging (Centeno et al., 2009; Dayod et al., 2010; Pravina et al., 2013). Increasing Ca accumulation in wheat grains is thus an important goal in wheat breeding.

Several studies on different plant species and crops identified putative quantitative trait loci (QTL) for calcium in grains of wheat, rice, sorghum, barley, maize, pearl millet, or beans (Peleg et al., 2009; Zhang et al., 2009; Goel et al., 2011; Orazaly et al., 2015; Fedorowicz-Strońska et al., 2017; Sharma et al., 2017). In a tetraploid wheat population of recombinant inbred lines (RILs), derived from a cross between durum wheat and wild emmer, nine significant QTLs were associated with calcium concentration in grains (Peleg et al., 2009). Goel et al. (2011) reported that 31 genes are responsible for calcium accumulation in rice and 28 genes in sorghum. Five QTLs were identified in *Arabidopsis thaliana*, in which they explained 36.4% of the variation in calcium content (Vreugdenhil et al., 2004).

European countries are among the top wheat producers and exporters in the world (FAO, 2017)^1^; thus we chose a panel of recent European wheat varieties to explore the genetic variation of calcium in 353 varieties [339 winter wheat (WW) and 14 spring wheat (SW) varieties], and to identify QTLs associated with this trait by using a genome-wide association study (GWAS) in order to detect potential candidate genes.

## Materials and Methods

### Plant Materials and Growth Conditions

A European wheat panel consisting of 353 varieties mainly coming from Germany and France was used in this study. This panel included 339 WW and 14 varieties of SW. Field experiments were carried out at the Leibniz Institute of Plant Genetics and Crop Plant Research in Gatersleben, Germany (51°490N, 11°170E, 112 m), during two consecutive seasons (2014/2015 and 2015/2016). The individual plot size was 1 m × 1.5 m with four rows spaced 0.20 m apart. All varieties were sown in autumn and subjected to standard agronomic wheat management practices.

### Determination of Calcium Concentrations

Phenotypic analysis was conducted for the whole set of wheat varieties in each season. For each sample, 50 kernels were counted using a digital seed analyzer/counter Marvin (GTA Sensorik GmbH, Neubrandenburg, Germany) and the thousand-grain weight (TGW) was estimated. The samples were milled using a Retsch mill (MM300, Germany) and the milled samples were dried overnight at 40°C. Calcium concentrations were measured by inductively coupled plasma optical emission spectrometry (ICP-OES, iCAP 6000, Thermo Fisher Scientific, Germany) combined with a CETAC ASXPRESS^TM^ PLUS rapid sample introduction system and a CETAC autosampler (CETAC Technologies, Omaha, United States). Fifty micrograms of dried and ground samples from each variety were wet digested in 2 ml nitric acid (HNO_3_, 69%, Bernd Kraft GmbH, Germany) using a high-performance microwave reactor (UltraClave IV, MLS, Germany). Digested samples were filled up to 15 ml final volume with de-ionized distilled water (Milli-Q^®^ Reference System, Merck, Germany). Element standards were prepared from Bernd Kraft multi-element standard solution (Germany). Calcium as an external standard and Y (ICP Standard Certipur^®^, Merck, Germany) were used as internal standards for matrix correction.

### Phenotyping and Statistical Analysis

The resulting calcium values for wheat grains of each variety and environment were used to calculate the best linear unbiased estimates (BLUEs), by applying the residual maximum likelihood (REML) algorithm with mixed linear models (MLMs) function (Yu et al., 2006) and considering genotype as fixed effect and environment as random effect. These calculations were accomplished using GenStat v16 software (VSN International, Hemel Hempstead, Hertfordshire, United Kingdom).

The broad sense heritability of Ca was calculated using the equation:

(1) $$\begin{array}{c} H^{2}\;=\;\sigma_{G}^{2}/(\sigma_{G}^{2}\;+\;(\sigma_{e}^{2}/nE) \end{array}$$

where $\sigma_{G}^{2}$ is the variance of the genotype, $\sigma_{e}^{2}$ represents the variance of the residual and nE is the number of the environments.

Analyses of variance (ANOVA) and Pearson’s correlation coefficient were calculated for the calcium trait across the two environments with SigmaPlot package 13.

### Genotyping

All wheat varieties were genotyped by the company Trait Genetics GmbH, Gatersleben, Germany^2^ using a new 90k iSELECT Infinium array (Wang et al., 2014) which contained 7761 mapped polymorphic single-nucleotide polymorphism (SNP) markers and a 35k Affymetrix-SNP array (Axiom^®^ Wheat Breeder’s Genotyping Array^3^) which contained 7762 mapped polymorphic SNPs. For the reference map, the ITMI-DH population (Sorrells et al., 2011; Poland et al., 2012) was used to anchor all SNP-markers. Only mapped markers with a minor allele frequency (MAF) ≥3% (equaling 11 varieties out of 353) were used for association analysis.

### GWAS Mapping and Linkage Disequilibrium Characteristics

GWAS analysis for the phenotypic and genotypic dataset was performed by using GenStat v16 software (VSN International, Hemel Hempstead, Hertfordshire, United Kingdom). Association analysis was performed using the “Single trait association analysis” function with Kinship matrix (K) as a relationship model to control for population structure by GenStat v16, though no obvious population structure was observed in the described population (Kollers et al., 2013).

To declare the significant marker–traits associations (MTAs), we considered a threshold *P*-value of -log_10_ (*P*) ≥3. When Bonferroni correction with *P* < 0.05 was applied the resulting -log_10_ (*P*) threshold rose to 5.49. The proportion of the phenotypic variation (*R*^2^) was calculated using the software package TASSEL 5.0. Marker effects (positive/negative) were estimated by GenStat v16 based on the effect of specific allele in the varieties.

BLUEs of the trait, each variety and across the seasons (2015 and 2016) were calculated by applying the “mixed models REML” module with the “linear mixed models” of GenStat v16.

Linkage disequilibrium (LD) which is the non-random association between pairs of loci was studied in the whole panel, observed by using squared allele frequency correlation and calculated within each chromosome. Loci in the LD region were determined according to the squared allele frequency correlations (*r*^2^) and were considered to be in significant LD when *r*^2^ ≥0.2. LD plots were performed by GenStat (v16) to examine the average LD decay within each chromosome.

### Physical Mapping Resources of Wheat and Identification of Putative Candidate Genes

While in GWAS analysis the marker data were connected with the phenotypic data in order to identify significant MTAs, in this step we identified the flanking sequence of SNP markers defining significant associations with the calcium trait. Markers which were located in significant LD regions were obtained from the wheat 90k database (Wang et al., 2014) and 35k database (see text footnote 3). The wheat marker sequences were blasted on the wheat genome assembly IWGSC1 and POPSEQ (The International Barley Genome Sequencing Consortium, 2012) and the website of Ensemble Plants ^4^ to obtain their corresponding genes, transcripts, and gene identifiers (IDs). Related regions for these significant associations were anchored using the wheat sequence assembly. For the resulting gene IDs the Human-Readable Descriptions were selected to define annotated gene functions by ftp://ftpmips.helmholtz-muenchen.de/plants/wheat/IWGSC/genePrediction_v2.2. The whole set of marker sequences was blasted using the software package Geneious 10^5^ and the most significant hit was selected (Kearse et al., 2012). A similar strategy was applied to find a candidate gene for eyespot resistance in wheat (Zanke et al., 2017).

## Results

### Description of Phenotypic Data

**FIGURE 1:**
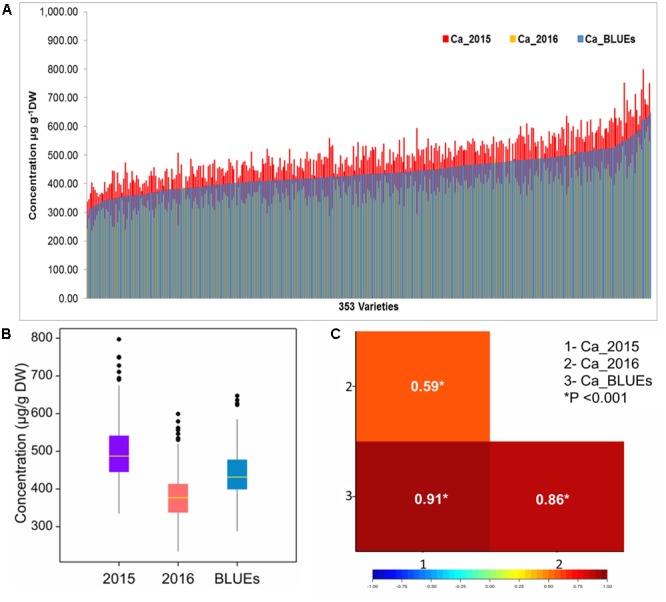
**(A)** Calcium concentration (μg g^-1^ DW) distribution among 353 European wheat varieties in two different seasons (2015 and 2016) and BLUEs. **(B)** Box plots of calcium values for two single season and BLUEs in 353 varieties. **(C)** Pearson correlation coefficients among (2015/2016) seasons and BLUE value.

**FIGURE 2:**
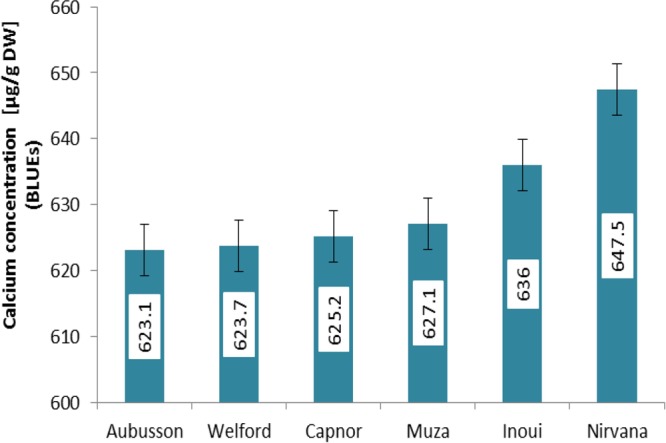
Calcium concentrations of the top 6 accessions [Ca > 600 (μg g^-1^ DW)] based on BLUE values ± SE. Numbers on the *y*-axis represent the calcium concentration.

Calcium measurements were performed for the whole set of European wheat varieties (WW = 339, SP = 14) grown in two seasons (2015 and 2016) (Supplementary Table S1). In each season calcium concentrations covered a wide range of variation (**Figure 1A**). In the season 2015, the highest measured grain Ca concentration was 797 μg g^-1^ DW, while in 2016 the highest Ca value was around 647 μg g^-1^ DW (**Figure 1B**). Estimated BLUEs ranged from 288.2 to 647.5 μg g^-1^ DW with a mean of 438.1 μg g^-1^ DW (**Figure 1B**). Based on BLUEs the highest scored value for calcium in the whole set of wheat varieties was 647.5 μg g^-1^ DW for the variety Nirvana from France (**Figure 2**). The ANOVA showed significant effects of the genotype and the environment on calcium concentrations in the grain (Supplementary Table S2). The Pearson’s correlation measured for calcium trait among the growing environments and BLUEs, ranged from 0.59 to 0.91 (*P* < 0.001, **Figure 1C**). The highest correlation was between season 2015 and BLUEs (*r* = 0.91, *P* < 0.01), while the lowest but still significant correlation was between seasons 2015 and 2016 (*r* = 0.59, *P* < 0.01). The broad sense heritability equaled 0.73 across the two environments for 353 varieties indicating that the phenotypic values in the two years are relatively stable for the different varieties.

### Detection of MTAs

**FIGURE 3:**
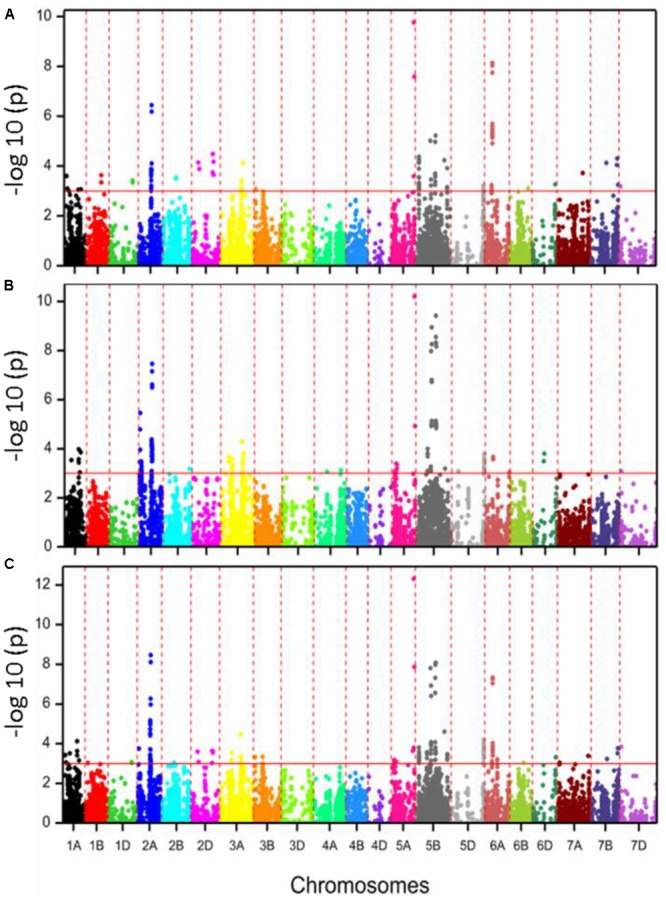
Manhattan plots with –log *P*-values of SNPs associated with calcium concentration values for 353 European wheat varieties based on 2015 **(A)**, 2016 **(B)**, and BLUE values **(C)**. **(A–C)** The red line color in the figure shows the threshold of –log_10_ (*P*-value) of three and all the significantly associated SNP markers are above the red line.

GWAS analysis was performed using a MLM with 90k and 35k SNP markers for the calcium data from the two growing seasons 2015 and 2016. Additionally the MTAs for the BLUEs from both years were calculated. Our analysis detected 485 significant [-log_10_ (*P*-value) ≥3] association signals for both environments and BLUEs (Supplementary Table S2). A number of 276 significant markers showed a positive allele effect, while the remaining markers (209 markers) had negative allele effects. These MTAs were located on all chromosomes except chromosomes 3D, 4B, and 4D (**Figure 3**). The most significant association was detected on chromosome 5A. On the other hand, most of the significant MTAs for grain calcium were identified on chromosome 2A (111 MTAs) and chromosome 5B (127 MTAs). On chromosome 2A, most of the MTAs were located in the genomic region of 64.3–67.4 cM. Based on the analysis, we found 31 consistent associations, which were present in both environments plus BLUEs and 20 consistent associations are above the Bonferroni correction threshold which equals 5.49 (**Table 1**). The explained phenotypic variances (*R*^2^) ranged from 0.81 to 11.27%.

**Table 1:** Summary of consistently significant markers detected in the two environments and BLUEs.

Marker^∗^	Chromosome	Position (cM)	-log_10_ (*P*) BLUEs	Effect BLUEs	% *R*^2^ BLUEs
BS00049644_51	2A	66.6	8.46	–20.89	4.60
RAC875_c24517_558	2A	64.3	4.73	–15.17	1.68
Kukri_c40035_258	2A	64.3	4.98	–15.60	1.85
AX-95169653	2A	66.6	6.27	18.27	3.12
AX-94940052	2A	66.6	5.97	–17.96	1.61
AX-94536561	2A	66.6	8.11	–20.42	3.44
AX-94881950	2A	64.3	5.17	–16.02	2.25
AX-94850365	2A	64.3	5.06	–15.85	2.19
AX-94560505	2A	64.3	4.42	–14.61	1.70
AX-94544896	2A	64.3	4.53	–14.78	1.78
AX-94404038	2A	64.3	4.96	–15.55	2.13
RFL_Contig1175_354	3A	109	4.47	19.60	0.81
wsnp_Ex_c20899_30011827	5A	117.7	7.87	45.80	7.83
RAC875_c8642_231	5A	114.5	12.31	43.28	11.27
AX-95077733	5A	117.7	7.87	45.80	8.26
snp_CAP8_c1210_739429	5B	149.8	4.61	–33.57	1.86
CAP7_c5481_96	5B	149.8	4.61	–33.57	1.86
RAC875_c30011_426	5B	78.7	6.93	–18.44	3.44
BS00062731_51	5B	78.7	6.41	–17.70	1.03
GENE-0168_7	5B	75.5	7.81	–21.80	4.67
AX-94644169	5B	103.2	8.08	–21.88	4.43
AX-94541836	5B	101.7	7.98	–20.14	2.80
AX-94547820	5B	100.9	7.32	–18.95	3.61
AX-94452355	5B	100.9	6.56	–17.89	2.40
Jagger_c8037_96	5D	167	3.87	13.73	1.64
wsnp_Ex_c17575_26300030	6A	37.3	7.27	24.98	3.55
wsnp_Ex_c17575_26299925	6A	37.3	7.33	25.12	3.72
Tdurum_contig62141_496	6A	37.3	7.27	24.98	3.55
Kukri_rep_c104648_439	6A	37.3	7.27	24.98	3.55
Kukri_c35661_63	6A	37.3	7.27	24.98	3.55
AX-94415776	6A	37.3	7.04	24.69	3.88

*^∗^Consistently significant markers appeared in the two environments (2015 and 2016) and BLUE values with threshold of -log_*10*_ (*P*-value ≥3). The highlighted markers with gray color are above the Bonferroni threshold which equals 5.49.*

**FIGURE 4:**
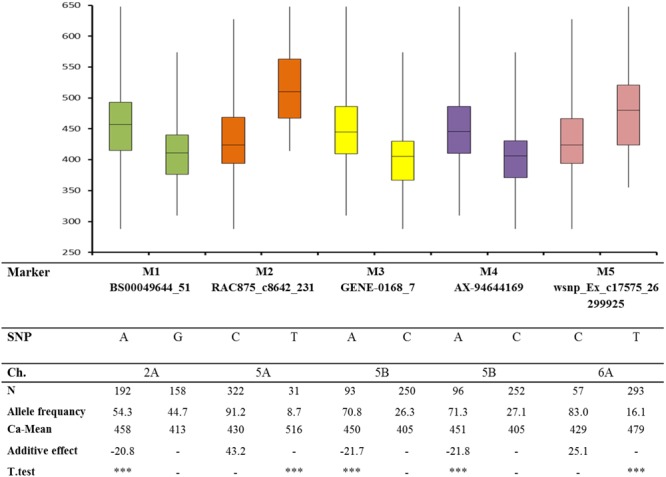
Box plot analysis for five significant markers based on BLUEs for calcium trait (MAF = AF). ^∗∗∗^denotes 0.001.

The additive effects of five representative significant markers based on BLUEs are depicted in **Figure 4**. Two significant markers [RAC875_c8642_231 marker (M2) (MAF = 0.09) and wsnp_Ex_c17575_26299925 marker (M5) (MAF = 0.16)] had positive allele effects. Another three significant markers [BS00049644_51 (M1), GENE-0168_7 (M3), and AX-94644169 (M4)] showed negative effects. Marker RAC875_c8642_231 (M2) had a highly significant positive effect (*P* < 0.001) with 43.3 μg g^-1^ DW (**Figure 4**).

### Connection of the Significant Markers to the Wheat Genome Sequence and Identification of Candidate Genes

In order to identify potential candidate genes for calcium concentrations in wheat grains, the significant SNP markers [–log_10_ (*P*-value) ≥3], together with other markers in LD (*r*^2^ ≥0.2), were used to query the available wheat genome sequence data in the POPSEQ assembly (Supplementary Table S4). The rationale behind this strategy was that the available DNA chips did not cover all genes in the wheat genome and that a highly significant marker may either be the causative gene itself or in close linkage to the causative gene.

Blast analysis of these markers using POPSEQ showed that chromosomes 2A, 5A, 5B, 5D, and 6A harbored many calcium- transporting genes. The most significant SNP (RAC875_ c8642_231) in our analysis was located on chromosome 5A (114.5 cM). The gene underlying this marker encodes a cation/sugar symporter, while the second significant locus (wsnp_Ex_c20899_30011827) on the same chromosome (117.7 cM) carries a gene that encodes an AP2-type transcription factor. We further detected two genes, which may be related to calcium transport near this significant region (114.5–117.7 cM): one gene (Traes_5AL_898DAA873) is related to plasma membrane ATPases while another gene (Traes_5AL_637EB761F) encodes an H^+^-ATPase. Furthermore, in the same region, we found a gene (Traes_5AL_AE6B41A0A) related to divalent metal cation transport together with two further genes (Traes_5AL_6C9A5537F and Traes_5AL_6C8BD96CB) related to heavy metal transport/detoxification. Along this chromosome we were able to find loci associated with Ca-permeable ion channels, such as Traes_5AL_E1F7DD9EA and Traes_5AL_C89AC9640 coding for cyclic nucleotide-gated channels (CNGCs) or Traes_5AL_F1522B81F and Traes_5AL_98814295D encoding mechanosensitive ion channels. However, these genes were not closely located to the significant markers (Supplementary Table S4).

In the LD region of chromosome 2A, we found a number of genes, which are related to calcium transport functions, such as Traes_2AL_72F83E7B0 and Traes_2AL_6069A8864 (mechanosensitive ion channel family protein), Traes_2AL_ D33454518 (cation/H^+^ antiporter), and Traes_2AL_CF9F964E6 underlying a calcium-transporting ATPase. The most significant association on chromosome 5B was the locus Traes_5BL_DF8D1B819, which is related to an ammonium transporter. Other closely linked genes are Traes_5BS_4AEE5C2AE encoding a mechanosensitive ion channel family protein and Traes_5BS_98C73F5CA, Traes_5BS_06F7D0060, Traes_5BS_272FDBF9D, Traes_5BL_4AACBDDAA, and Traes_5BL_E4BE45756, all related to cation/H^+^ antiporters. Near to the highly significant markers of chromosome 5B (depicted in yellow in Supplementary Table S4) a gene for a CNGC (Traes_5BL_411EF97B9) is located, while a cation/calcium exchanger (Traes_5BL_6A7BE3F0C) is located quite distantly from the significant markers and are therefore not likely as candidate genes.

## Discussion

### European Wheat Germplasm Harbors a Large Genotypic Variability in Calcium Accumulation

Genetic fortification strategies are highly suitable for developing wheat varieties with high mineral element contents. Therefore, this study focused on investigating the natural genetic variation in European wheat varieties and on identifying candidate genes contributing to calcium accumulation in wheat grains. Phenotypic analysis for calcium concentrations showed a wide variation between the varieties based on BLUEs which ranged from 288.2 to 647.5 μg g^-1^ DW. The heritability was high (0.73) indicating that the major part of the variability was due to genotypic effects, which is in agreement with previous studies (Vreugdenhil et al., 2004; Garcia-Oliveira et al., 2009; Peleg et al., 2009). Very strong, significant correlation coefficients were detected between the two seasons indicating that the phenotypic measurements were quite stable in the different years. This conclusion was also supported by a high heritability. Considering the analysis across the two growth seasons, the results showed that genotypic variances due to genotypes were significant (at *P* < 0.01). Marker effects (*R*^2^) which explained the proportion of phenotypic variance for consistently significant markers (appearing significant in both seasons and BLUEs) contributed a modest proportion ranging from 0.81 to 11.27%. The ANOVA results indicated that genotypes and environmental factors have a significant effect on calcium concentration in wheat grains. A similar conclusion was reached by (Gu et al., 2015) for grain Ca in maize.

### Calcium Accumulation in Grains Is Controlled by Multiple Loci

In the present study, genome mapping revealed that most of the significant MTAs for the consistently significant markers in 2015, 2016, and BLUEs (**Table 1**) are conferred mostly by genome A (chromosomes 2A, 3A, 5A, and 6A), while one locus was related to the B genome (chromosome 5B) and another one related to the D genome (chromosome 5D). A mapping study of a RIL population in tetraploid wheat detected significant QTL for calcium concentration on chromosomes 1A, 4A, 6A, 2B, 4B, 5B, 6B, and 7B (Peleg et al., 2009). Another study on bread wheat for calcium-dependent protein kinases (CDPKs) which are crucial sensors of calcium concentration changes in plant cells, identified 20 CDPK genes (Li et al., 2008). To our knowledge, this is the first report on GWAS for grain calcium concentration in hexaploid wheat. Thus, further genetic and functional analysis of associated genomic regions may shed further light on the genetic basis of improved calcium concentration in wheat grains.

### Putative Candidate Genes for Ca-related QTLs

In general, calcium transporters are involved in the cellular compartmentalization of calcium in different plant organs. Three major gene families of calcium transporter proteins have been described: (i) Ca^2+^-transporting P-type-ATPases [endoplasmic reticulum-type Ca^2+^-ATPase (ECA/IIA Type) and autoinhibited Ca^2+^-ATPase (ACA/IIB-type)], (ii) divalent cation–H^+^ antiporters/exchangers [cation/H^+^ antiporters (CAX), CCX and CHX], and (iii) Ca-permeable ion channels that include mechanosensitive calcium-permeable channels (MSCCs), glutamate receptors (GLRs), CNGCs and two-pore channels (TPC) (Vinoth and Ravindhran, 2017). The highly significant SNP-markers (**Table 1** and Supplementary Table S3) could either be derived from the causative genes themselves or be in linkage to the causative genes for the identified QTLs for Ca. In **Table 2**, we compiled the list of Ca-related genes or transporters which are in close vicinity to highly significant SNP-markers and which are therefore potential candidate genes for the Ca-related QTLs.

**Table 2:** Putative candidate genes for Ca in the vicinity of highly significant associated SNP-marker.

Significant marker	Chromosome,	(*r*^2^)	–log_10_(*p*)	Effect	% *R*^2^	Gene name	Human_Readable_Description
	position (cM)	bw_SNP			BLUES	(IWGSC_v1)	
RAC875_c8642_231	5A, -114.5	0.46	12.31	43.72	11.27	Traes_5AL_F49663738	Sugar transporter/solute-cation symport
wsnp_Ex_c20899_30011827	5A, -117.7	1.00	7.87	46.14	7.83	Traes_5AL_19637DE03	AP-2 complex subunit alpha-1
–	5A	–	–	–	–	Traes_5AL_6C8BD96CB	Heavy metal transport/detoxification superfamily protein
–	5A	–	–	–	–	Traes_5AL_898DAA873	Plasma membrane ATPase 1
–	5A	–	–	–	–	Traes_5AL_637EB761F	H(+)-ATPase 11
–	5A	–	–	–	–	Traes_5AL_AE6B41A0A	Divalent metal cation transporter MntH
–	5A	–	–	–	–	Traes_5AL_6C9A5537F	Heavy metal transport/detoxification
–	5A	–	–	–	–	Traes_5AL_6C8BD96CB	Heavy metal transport/detoxification
AX-94940052	2A, -66.6	0.48	5.97	–17.96	1.61	Traes_2AL_156C770EE	Receptor-like protein kinase 2
–	2A	–	–	–	–	Traes_2AL_542E74269	Potassium channel AKT1
Kukri_c40035_258	2A, -64.0	0.10	4.98	–15.60	1.85	Traes_2AS_0C87833C6	Phosphatidylinositol-4-phosphate 5-kinase family protein
–	2A	–	–	–	–	Traes_2AL_72F83E7B0	Mechanosensitive ion channel family protein
–	2A	–	–	–	–	Traes_2AL_6069A8864	Mechanosensitive ion channel family protein
–	2A	–	–	–	–	Traes_2AL_D33454518	Cation/H^+^ antiporter
–	2A	–	–	–	–	Traes_2AS_B264257CD	Flavin-binding monooxygenase family protein
–	2A	–	–	–	–	Traes_2AL_CF9F964E6	Calcium-transporting ATPase
–	2A	–	–	–	–	Traes_2AS_95611CAD2	Heavy metal transport/detoxification superfamily
–	2A	–	–	–	–	Traes_2AL_6DD37E6BE	Heavy metal transport/detoxification superfamily
–	2A	–	–	–	–	Traes_2AL_9B175F3Da	Heavy metal transport/detoxification superfamily
–	2A	–	–	–	–	Traes_2AL_F360E3FE3	Heavy metal transport/detoxification superfamily
–	2A	–	–	–	–	Traes_2AL_13CBA4FEA	Heavy metal transport/detoxification superfamily
–	2A	–	–	–	–	Traes_2AS_AA84E72D4	Heavy metal transport/detoxification superfamily
–	2A	–	–	–	–	Traes_161086245	Heavy metal transport/detoxification superfamily
AX-94547820	5B, -100.9	0.03	7.20	–18.80	3.61	Traes_5BL_DF8D1B819	Ammonium transporter 2
–	5B	–	–	–	–	Traes_5BL_411EF97B9	Cyclic nucleotide-gated channel

Based on our investigations, we found 41 potential calcium-transporting genes distributed over six chromosomes (2A, 3A, 5A, 5B, 5D, and 6A). These include 8 Ca/proton exchangers, 4 Ca-ATPases, and 31 channels in addition to other genes that are putatively related to calcium transport, such as Traes_5AL_6C9A5537F which is annotated as heavy metal transporter (Supplementary Table S4). On chromosome 5A, the most significantly associated MTA with a *R*^2^ value equaling to 11.27% and a favorable additive effect, is related to Traes_5AL_F49663738 gene encoding a putative cation/sugar symporter. The second significant gene (Traes_5AL_19637DE03) with *R*^2^ equaling 7.83 encodes an AP-2 complex subunit alpha-2-like protein that is possibly related to calcium transport function (Matros et al., 2017). Another significant SNP-marked gene (Traes_5AL_320913F7A) also located on the same chromosome and related to a gene of the 2S albumin superfamily, which encodes as a storage protein (Yamazaki et al., 2008). All of these three genes are located on a region between 114.5 and 117.7 cM on chromosome 5AL. In addition to six Ca^+2^ channels associated with these markers: Traes_5AL_BBFBC2F48, Traes_5AL_B598F5A0D, Traes_5AL_E1F7DD9EA, Traes_5AL_C89AC9640, Traes_5AL_F1522B81F, and Traes_5AL_98814295D distributed along chromosome 5A (**Table 2** and Supplementary Table S4), we also detected on this chromosome, two genes (Traes_5AL_6C9A5537F and Traes_5AL_6C8BD96CB) that encode for heavy metal transport/detoxification superfamily proteins involved in metal ion binding (Hall, 2002). Near the significant region, the Traes_5AL_AE6B41A0A marker relates to divalent metal cation transporters that may also act as calcium transporter. Significant associations were also noted on chromosome 2A with 11 SNP markers located within this region (64–66.6 cM) and some of them encoding a disease resistance protein, CBS domain-containing protein, receptor-like protein kinase 2, phosphatidylinositol-4-phosphate 5-kinase family protein, NHL domain-containing protein or Rho GTPase-activating protein besides other genes with unknown function. The LD region on chromosome 2A is widely spread on the physical map of the genome assembly of IWGSC1 extending to the long and the short arm of chromosome 2A. Discrepancies in the order of the contigs in this genome assembly were already described in Zanke et al. (2017). This region contains a number of genes potentially related to calcium-accumulation such as mechanosensitive ion channel family proteins (Traes_2AL_6069A884, Traes_2AL_72F83E7B0) and a number of heavy metal transport/detoxification superfamily proteins (Traes_2AS_95611CAD2, Traes_2AL_6DD37E6BE, Traes_2AL_9B175F3Da, Traes_2AL_F360E3FE3, Traes_2AL_13CBA4FEA, Traes_2AS_AA84E72D4, and Traes_161086245). Nine significant SNPs occurred on chromosome 5B encoding for different functions and some of them may be involved in calcium transport, like Traes_5BL_DF8D1B819 gene which is located on 100.9 cM and is encodes an ammonium transporter. On chromosome 5D, there were two significant markers: Jagger_c8037_96 and BS00032035_51 with unknown functions. On chromosome 6A are located six significant SNP markers, which are related to two genes encoding histone superfamily proteins with a role in the activation of calcium/calmodulin-dependent protein kinases (Davis et al., 2003). Based on our results, the annotated functions of significant genes and genes in the LD region suggested the presence of several genes controlling the calcium uptake. These genes can be considered as putative candidate genes for calcium accumulation in wheat grains and provide a solid resource for future work. However, further functional validation of these genes and their role in calcium uptake in wheat grains is still needed.

## Conclusion

Apart from focusing on the concentrations of iron and zinc in wheat, which has taken much attention in previous studies, only few genetic studies are available on calcium concentrations in wheat grains are available. Improving levels of grain calcium concentration in hexaploid wheat remains one of the most important breeding objectives for the nutritional security of the whole population and especially for the poor from the nations where wheat is the main source of calories. Overall, through measurable phenotypic and genotypic variation for grain calcium concentrations as well as by considering the detected favorable QTLs distributed across various chromosomes and potentially responsible genes in the current research, we aimed to deepen the understanding of the genetic basis of calcium accumulation in wheat grains and to open the door to more efficient ways to increase calcium concentration in the grain and thereby overall wheat quality.

## Author Contributions

DA performed the data analysis including genome-wide association scan and related analyses. KE and NvW participated in calcium concentration measurements. KP and MR designed the experiment. MR conceived the idea and participated in the interpretation of results. DA and MR wrote the manuscript. All authors read and approved the final manuscript.

## Conflict of Interest Statement

The authors declare that the research was conducted in the absence of any commercial or financial relationships that could be construed as a potential conflict of interest.

## Footnotes

1 http://www.fao.org/worldfoodsituation/csdb/en/

2 www.traitgenetics.com

3 http://www.cerealsdb.uk.net/

4 http://plants.ensembl.org/Triticum_aestivum/Tools/Blast

5 http://www.geneious.com
