# Supplementary material for: Genome-Wide Association Study of Calcium Accumulation in Grains of European Wheat Cultivars
Source: Front Plant Sci. 2017 Oct 27;8:1797. doi: 10.3389/fpls.2017.01797 (PMC5663994; doi:10.3389/fpls.2017.01797)
Supplement: Supplementary file 2 [file Table_2.docx]

**Supplementary table S2: Analysis of variance (ANOVA) of Calcium concentration in 353 varieties among two environments.**

| **Source of Variation** | **DF** | **SS** | **MS** | **F** | **P** |
| --- | --- | --- | --- | --- | --- |
| **Genotype** | 353 | 2619640.45 | 7421.07 | 3.744 | <0,001 |
| **Environment** | 1 | 2490768.45 | 2490768.45 | 1256.49 | <0,001 |
| **Residual** | 353 | 699759.68 | 1982.32 |  |  |
| **Total** | 707 | 5810168.59 | 8218.06 |  |  |

DF = Degrees of freedom

SS = Sum of Squares

MS = Mean of Squares

* P<0.001
